# Tissue Factor Residues That Putatively Interact with Membrane Phospholipids

**DOI:** 10.1371/journal.pone.0088675

**Published:** 2014-02-06

**Authors:** Ke Ke, Jian Yuan, James H. Morrissey

**Affiliations:** Department of Biochemistry, University of Illinois, Urbana, Illinois, United States of America; Emory University School of Medicine, United States of America

## Abstract

Blood clotting is initiated by the two-subunit enzyme consisting of the plasma protease, factor VIIa (the catalytic subunit), bound to the integral membrane protein, tissue factor (the regulatory subunit). Molecular dynamics simulations have predicted that certain residues in the tissue factor ectodomain interact with phosphatidylserine headgroups to ensure optimal positioning of the tissue factor/factor VIIa complex relative to its membrane-bound protein substrates, factors IX and X. In this study, we individually mutated to alanine all the putative phosphatidylserine-interactive residues in the tissue factor ectodomain and measured their effects on tissue factor cofactor function (activation of factors IX and X by tissue factor/factor VIIa, and clotting of plasma). Some tissue factor mutants exhibited decreased activity in all three assays, with the most profound defects observed from mutations in or near the flexible loop from Lys159 to Gly164. The decreased activity of all of these tissue factor mutants could be partially or completely overcome by increasing the phosphatidylserine content of tissue factor-liposomes. Additionally, yeast surface display was used to screen a random library of tissue factor mutants for enhanced factor VIIa binding. Surprisingly, mutations at a single amino acid (Lys165) predominated, with the Lys165→Glu mutant exhibiting a 3-fold enhancement in factor VIIa binding affinity. Our studies reveal the functional contributions of residues in the C-terminal half of the tissue factor ectodomain that are implicated in interacting with phosphatidylserine headgroups to enhance tissue factor cofactor activity, possibly by allosterically modulating the conformation of the adjacent substrate-binding exosite region of tissue factor.

## Introduction

Tissue factor (TF) is the cell-surface receptor for factor VIIa (FVIIa), with the resulting TF/FVIIa complex activating factors IX (FIX) and X (FX) by limited proteolysis [1]. Binding of FVIIa to membrane-anchored TF has multiple effects on enzyme activity: (a) TF allosterically activates FVIIa, independent of the membrane [2]; (b) TF is thought to contribute a substrate-binding site (exosite) to help recognize FIX and FX [3]–[6]; (c) phosphatidylserine (PS) dramatically increases rates of FIX and FX activation by TF/FVIIa [7]; and (d) TF fixes FVIIa’s active site at a set distance above the membrane surface, which may be important for optimal engagement of the scissile bonds of FIX and FX [8]–[11].

The precise nature of the interactions between TF, FVIIa and its substrates on the membrane are not unknown. Molecular dynamics simulations of the TF ectodomain (sTF) on the membrane surface identified direct interactions between certain TF residues and PS headgroups [10]. Interestingly, the TF residues predicted to interact with PS are located very close to the substrate-binding exosite region of this protein. This prompted us to hypothesize that PS-interacting residues in the TF ectodomain are in allosteric linkage with the nearby exosite region, possibly promoting a conformation better able to bind FIX and FX.

In this study, we used mutagenesis to examine how TF residues predicted to interact with PS contribute to the proteolytic activity of the TF/FVIIa complex. We also selected TF mutants with enhanced binding affinity to FVIIa. Our findings are consistent with the notion that conformational changes in TF, induced upon binding to both FVIIa and PS, may help regulate the catalytic activity of TF/FVIIa on membrane surfaces.

## Materials and Methods

### Materials

Materials were from the following suppliers: pooled normal human plasma, George King Bio-Medical (Overland Park, KS, USA); porcine brain PS, egg phosphatidylcholine (PC) and egg phosphatidylethanolamine (PE), Avanti Polar Lipids (Alabaster, AL, USA); recombinant human FVIIa, American Diagnostica (Greenwich, CT, USA); human plasma FIX and FX, Enzyme Research Laboratories (South Bend, IN, USA); and fluorescein-Phe-Pro-Arg-chloromethylketone (*fl-*FPRck), Haematologic Technologies, Inc. (Essex Junction, VT, USA). *Fl*-FVIIa was prepared by reacting FVIIa to completion with *fl*-FPRck (confirmed by loss of activity), after which excess inhibitor was removed by dialysis.

### Recombinant Membrane-anchored TF (membTF)

MembTF (amino acids 3–244) was expressed in *E. coli* and purified as described [12]. MembTF was incorporated into liposomes using Bio-Beads® SM-2 and 20 mM sodium deoxycholate [13], using a membTF:phospholipid molar ratio of 1∶8700. Concentrations of available TF on liposomes are reported here and were quantified by FVIIa titration as described [14].

### FVIIa Amidolytic Activity

Initial rates of FVIIa-catalyzed hydrolysis of Chromozym t-PA substrate (Roche Applied Science, Indianapolis, Indiana, USA) were quantified as described [11]. Reaction conditions were: 5 nM FVIIa, 100 nM membTF, 1 mM Chromozym t-PA in HBSAC (25 mM HEPES pH 7.4, 100 mM NaCl, 5 mM CaCl_2_, 0.1% BSA, 0.1% NaN_3_) with 0.1% Triton X-100.

### FX Activation

Initial rates of FX activation by TF/FVIIa were typically quantified in a two-stage assay as described [15], with the following modifications. For assays using liposomes, FVIIa and relipidated membTF were incubated for 5 min in HBSAC, then FX was added. Reactant concentrations were: 100 pM FVIIa, 2 nM membTF and 100 nM FX for membTF-liposomes without PS; or 5 pM FVIIa, 100 pM membTF and 100 nM FX for membTF-liposomes with PS. Timed 10 µL aliquots were removed over 10 min and quenched in 25 µL Stop Buffer (60 mM Mes-NaOH pH 5.8, 75 mM EDTA, 50 mM NaCl, 8% Lubrol PX). FXa concentrations were measured the change in A_405_ following addition of 10 µL 2.0 M Tricine-NaOH (pH 8.4) and 50 µL 1 mM Spectrozyme FXa substrate (Sekisui Diagnostics, Stamford, Connecticut, USA). For assays in solution (no phospholipids), initial rates of FX activation were measured in HBSAC buffer with 0.1% Triton X-100, using 5 nM FVIIa, 100 nM membTF, 100 nM FX, and 0.5 mM Spectrozyme FXa. For assays using membTF in solution or membTF-liposomes, rates of FXa generation in the presence of membTF mutants were typically normalized to those measured in the presence wild-type membTF with the same phospholipid composition.

Kinetic constants for FX activation were measured using a single-stage assay as described [11], with the following modifications. FVIIa and relipidated membTF were preincubated for 10 min, after which FX and Spectrozyme FXa were added and the change in A_405_ was measured. Reactant concentrations were: 2 nM FVIIa, 100 pM membTF and 0.6–9 µM FX for membTF-liposomes without PS; 2 nM FVIIa, 25 pM membTF and 0.1–1.5 µM FX for membTF-liposomes with 5% PS; or 2 nM FVIIa, 5 pM membTF and 0.02–0.3 µM FX for membTF-liposomes with 20% or 30% PS.

### FIX Activation

Initial rates of FIX activation by FVIIa bound to relipidated membTF were quantified by a modification of the two-stage method for FX activation. Reactant concentrations were: 5 nM FVIIa, 50 nM membTF and 800 nM FIX for membTF-liposomes without PS; or 150 pM FVIIa, 1.5 nM membTF and 800 nM FIX for membTF-liposomes with PS. Timed 40 µL aliquots were sampled over 40 min and quenched in 20 µL Stop Buffer. FIXa concentrations were measured by adding, to the quenched reactions, 10 µL 2.0 M Tricine-NaOH (pH 8.4) and 50 µL CBS 31.39 substrate (Diagnostica Stago, Parsippany, NJ, USA) in 33% ethylene glycol, and quantifying the rate of change in A_405_ [16]. Rates with membTF mutants were normalized to those with wild-type membTF, as with FX assays.

### Clotting Assay

Clotting assays of relipidated membTF were carried out in an ST4 coagulometer (Diagnostica Stago, Parsippany, NJ, USA) as described [17]. Specific activities of membTF mutants were compared by reference to a standard curve using wild-type membTF-liposomes of the same phospholipid composition.

### Yeast Surface Display

A random library of mutant sTF cDNAs (0.3% error rate) was prepared essentially as described [18], by generating random nucleotide errors using *Taq* polymerase (Invitrogen, Carlsbad, CA, USA) with 0.5 mM MnCl_2_ and 2 mM MgCl_2_, then cloning into pCT302 vector by homologous recombination. Expression of sTF on the yeast surface was confirmed by flow cytometry using anti-TF monoclonal antibody TF9-5B7 [19]. For selection, yeast were incubated with 8 nM *fl*-FVIIa for 40 min, washed briefly with HBSAC, and then sorted using a MoFlo cell sorter (DakoCytomation, Ft. Collins, CO, USA) [18], [20]. 5×10^7^ cells were screened, from which the ∼0.5% with the highest *fl*-FVIIa binding signal were collected. After four additional rounds of sorting with *fl*-FVIIa and growth, plasmids were rescued from isolated yeast clones and subjected to DNA sequencing. A representative sTF mutant containing the Lys165→Glu mutation was cloned in *E. coli*, expressed and purified as described [12].

### Surface Plasmon Resonance (SPR) Analyses

FVIIa binding affinities of mutant and wild-type sTF were determined at 25°C using a Biacore 3000 (GE Healthcare, Pittsburgh, PA, USA). HPC4 antibody was bound to CM5 sensorchips by standard amine coupling; after blocking and washing, HPC4-tagged sTF [21] was captured. FVIIa binding affinities for sTF were calculated from binding kinetics as described [22], with the following modifications. We determined *k*_off_ by analyzing response curves after saturation with 50 nM FVIIa. Association rate constants (*k*_s_) were calculated from four to six FVIIa concentrations, with *k*_on_ determined from the concentration dependence of *k*_s_. Kinetic constants were determined by nonlinear regression analysis as described [23]. Stand errors reported were from the plot fits.

## Results and Discussion

### Effects of TF Mutations on FVIIa-dependent Activation of FIX and FX

Based on previous molecular dynamics simulations of interactions between phospholipid bilayers and sTF [10], twenty surface-exposed residues in the C-terminal half of the TF ectodomain were selected for mutagenesis. This included all eighteen residues identified as interacting with PS, plus adjacent residues G164 and K165 (listed in Table 1). The TF mutants and wild type were expressed as recombinant membrane-anchored human TF (membTF), purified and incorporated into liposomes containing 0 to 30% PS (balance = PC). FVIIa was then added and initial rates of FIX and FX activation were measured. Table 1 reports normalized FX activation rates for all twenty mutants in 5% PS/95% PC liposomes, and also summarizes results from previously published studies of these mutants, where available. Twelve of the mutants (Q118A, V119A, G120A, T121A, K122A, S161A, K181A, G182A, E183A, N184A, Q212A and E213A) supported FX activation rates that were ≥70% of that of wild-type, and were therefore not studied further. To our knowledge, the effects of mutating these twelve residues on TF/FVIIa proteolytic activity have not previously been reported, except for mutation of residue S161 [24].

**Table 1:** Ability of membTF mutants to support activation of FX and FIX, and clotting of plasma.

	FX activation		FIX activation		Clotting activity	
TF Mutant	This study^a^	Literature	This study^a^	Literature	This study^a^	Literature
Q118A	97%		–b		–b	
V119A	98%		–b		–b	
G120A	101%		–b		–b	
T121A	90%		–b		–b	
K122A	93%		–b		–b	
**K159A**	24±3.2%	<30%^c^; ∼100%^d^	28±2.9%	30–75%^c^	3±1%	8%^e^; ∼5%^d^
**S160A**	66±3.6%		58±5.1%		24±8%	95%^e^
S161A	114%		–b		–b	128%^d^
**S162A**	53±1.3%		54±4.4%		16±3%	82%^d^
**S163A**	5±0.7%	<30%^c^	8.7±1.1%	<30%^c^	0.20±0.08%	11%^d^
**G164A**	5±0.5%	<30%^c^	10±1.4%	<30%^c^	0.20±0.10%	3%^d^
**K165A**	14±0.7%	17%^f^; <30%^c^	21±1.8%	<30%^c^	1.2±0.05%	30%^g^
**K166A**	6.4±0.5%	5%^f^; <30%^c^	14±1.5%	<30%^c^	0.30±0.09%	12%^g^
**D180A**	64±10%		67±15%		20±8%	
K181A	139%		–b		–b	
G182A	82±3.8%		–b		–b	
E183A	137%		–b		–b	
N184A	79±6.0%		–b		–b	
Q212A	87±9.3%		–b		–b	
E213A	82±12%		76±9.3%		–b	

a Data are mean ± standard deviation of normalized rates of FX and FIX activation, and clotting activities, using membTF mutants in 5% PS/95% PC vesicles. Eight mutants selected for study are boldfaced.

b Not determined.

c Kirchhofer et al. [3], using sTF with either SW-13 cell membranes or 500 µM 70% PS/30% PC liposomes.

d Ruf et al. [24], using detergent lysates of cells transfected to express membrane-anchored TF.

e Rehemtulla et al. [32], using detergent lysates of cells transfected to express membrane-anchored TF.

f Roy et al. [30], using detergent lysates of cells transfected to express membrane-anchored TF, diluted in a buffer with 100% PC vesicles.

g Ruf et al. [31], using detergent lysates of cells transfected to express membrane-anchored TF.

We next examined, in detail, the properties of the eight membTF mutants whose FX activation rates were <70% of wild-type (listed in boldface in Table 1). We first tested the ability of these membTF mutants to bind FVIIa in solution (i.e., with membTF dissolved in nonionic detergent) and found that all eight supported wild-type levels of FVIIa amidolytic activity (Table 2). This finding demonstrates that these membTF mutants bind FVIIa normally and exhibit normal allosteric activation of FVIIa, as revealed by hydrolysis of a tripeptidyl substrate. On the other hand, all the mutants exhibited substantially reduced abilities to support FX activation by FVIIa, even when tested in solution (Table 2). We note that these eight membTF mutants exhibited comparable or greater reductions in activity as measured in solution, compared to when the mutants were incorporated into liposomes with 5% PS (Table 1). Therefore, the reduction in TF cofactor activity caused by these mutations is not dependent on the presence of the phospholipid membrane surface.

**Table 2:** Ability of membTF mutants to support FVIIa amidolytic activity and FX activation in solution (with 0.1% Triton X-100).

TF mutant	FVIIa amidolytic activity	FX activation
K159A	101±3.1%	15±1.1%
S160A	104±4.4%	30±1.8%
S162A	106±4.1%	35±3.2%
S163A	105±3.6%	2.3±0.4%
G164A	104±4.9%	1.7±0.2%
K165A	106±4.1%	6.1±0.3%
K166A	107±3.4%	3.6±0.1%
D180A	99±2.3%	69±5.4%

**Figure 1:**
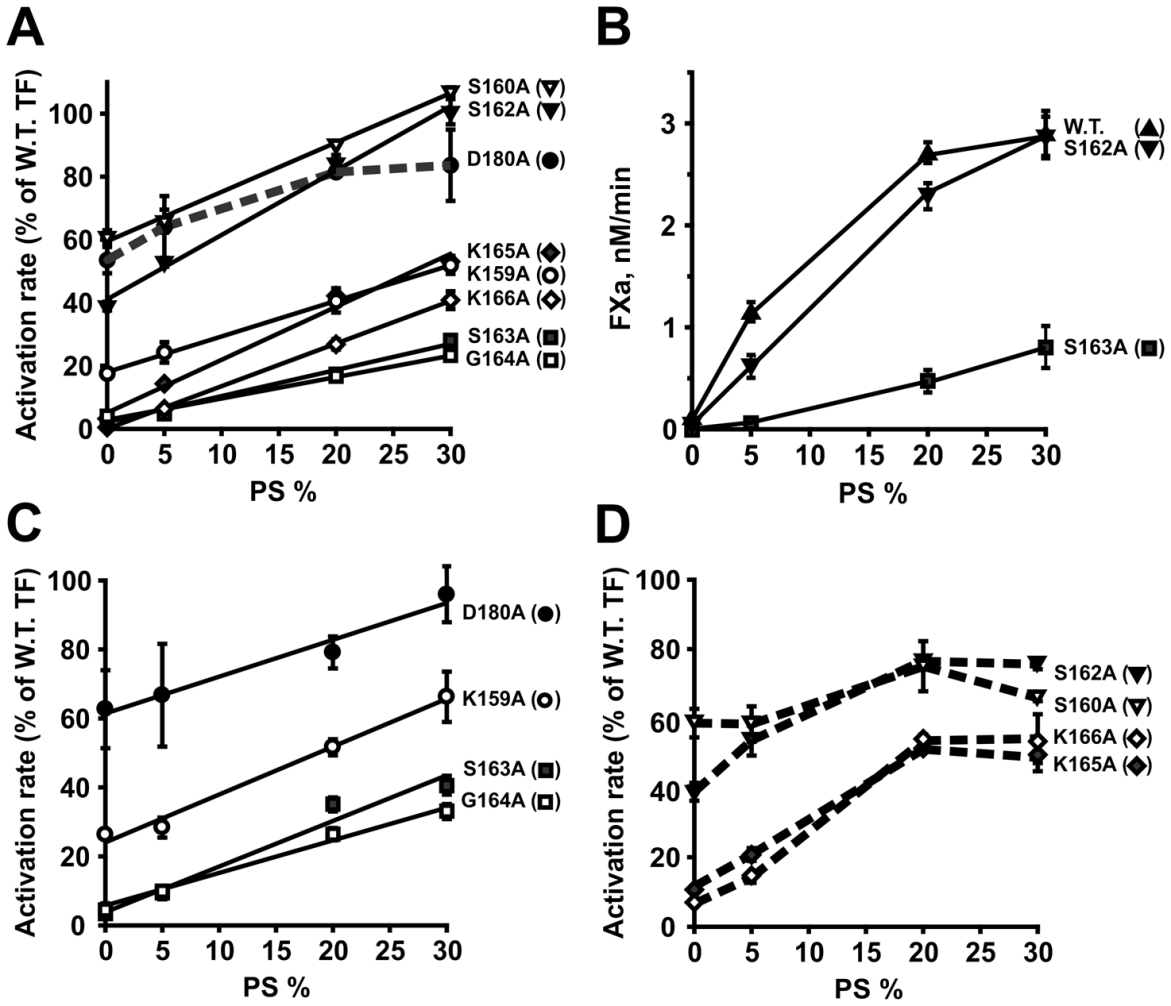
PS dependence of the effects of membTF mutations on FX and FIX activation. Initial rates were quantified for activation of FX (A,B) or FIX (C,D) by FVIIa bound to membTF mutants in liposomes of the indicated mol% PS (balance = PC). Data in panels A, C and D are normalized to those obtained with wild-type membTF at the same PS content. *Solid lines* were plotted if they fit the data with an r^2^≥0.95. *Dashed lines* connect data points if an attempt to fit a line yielded an r^2^<0.95. For clarity, the *solid* and *dashed lines* for FIX activation are plotted in separate panels (C and D). In panel B, the absolute initial rates were plotted for FX activation by FVIIa bound to wild-type membTF (W.T.) and two mutants (S162A and S163A). Data in all panels are mean ± SD, *n = *3.

When we incorporated the membTF mutants into liposomes containing either 100% PC or 5% PS/95% PC, five of them (K159A, S163A, G164A, K165A, and K166A) supported FX activation rates that were <30% of wild type, while the other three (S160A, S162A, D180A) supported FX activation rates that were 30–70% of wild type (Figure 1A). The decreased FX activation rates supported by these mutants were ameliorated by further increasing the PS content of liposomes, with a linear relationship between activity and PS content from 0 to 30% PS (except for D180A, Figure 1A). Since these rates are normalized to wild-type, our results indicate that PS enhances the cofactor activity of all eight mutants to an even greater degree for the mutants than for wild-type TF. Furthermore, mutants S162A and K165A exhibited steeper slopes than the others, so their activities are especially sensitive to increasing PS content. In order to better appreciate the differential effect of varying PS content on the catalytic activity of membTF/FVIIa complexes, Figure 1B shows absolute rates of FX activation supported by wild-type membTF and two representative mutants (S162A and S163A).

To our knowledge, the effects of five of these eight mutations on FX activation were previously tested by others (i.e., all but S160A, S162A and D180A; Table 1). There was reasonable agreement between our findings and previous studies with regard to rates of FX activation, with the exception of a single report indicating that K159A had ∼100% activity [24]. It should be noted, however, that the previous mutational studies of TF typically employed detergent lysates of transfected cells (see Table 1), so the membrane compositions were unknown and not controlled. An exception was the study of Kirchhofer *et al.* [3], but it used sTF and very high liposome concentrations, which also renders direct comparisons with our findings difficult.

Our data above were obtained using a single concentration of FX (100 nM), so the effects of the TF mutations on activity of the TF/FVIIa complex could be due to reduction in *k*_cat_, increase in *K_m_*, or a combination. To examine their effects on kinetics of FX activation by FVIIa, we focused on two membTF mutants that exhibited either a modest (S162A) or severe (S163A) reduction in TF cofactor function. We then measured the kinetic constants for FX activation by FVIIa bound either to wild-type or mutant membTF, incorporated into liposomes with a range of PS contents (Table 3). For the S162A mutant, the *k*_cat_ was somewhat reduced (i.e., from 34% of wild-type in 100% PC liposomes to 79% of wild-type in 30% PS liposomes). The *K_m_* showed a slight increase (1.2-fold) relative to wild-type in 100% PC liposomes, and essentially no difference from wild-type in 30% PS liposomes. Accordingly, the catalytic efficiency (*k*_cat_/*K_m_*) exhibited by the S162A mutant varied from 27% of wild-type in 100% PC liposomes to 78% of wild-type in 30% PS liposomes.

**Table 3:** Summary of kinetic constants for activation of FX.

	Wild-type			S162A			S163A		
% PS*	*k*_cat_	*K_m_*	*k*_cat_/*K_m_*	*k*_cat_	*K_m_*	*k*_cat_/*K_m_*	*k*_cat_	*K_m_*	*k*_cat_/*K_m_*
	min^−1^	µM	M^−1^s^−1^	min^−1^	µM	M^−1^s^−1^	min^−1^	µM	M^−1^s^−1^
0	16.4±2.5	4.42±0.71	6.2×10^4^	5.61±0.6	5.50±1.2	1.7×10^4^	0.872±0.11	7.42±0.30	2.0×10^3^
5	37.2±4.4	0.597±0.085	1.0×10^6^	17.7±3.0	0.811±0.029	3.6×10^5^	1.79±0.19	0.897±0.25	3.3×10^4^
20	111±18	0.103±0.008	1.8×10^7^	79.2±4.6	0.106±0.001	1.3×10^7^	14.6±0.69	0.120±0.005	2.0×10^6^
30	248±23	0.115±0.008	3.6× 10^7^	196±19	0.116±0.015	2.8× 10^7^	35.9±9.3	0.104±0.005	5.8× 10^6^

*Liposomes contained the indicated % PS (balance = PC).

For the S163A mutant, the reduction in *k*_cat_ ranged from 5.3% of wild-type in 100% PC liposomes to 14% of wild-type in 30% PS liposomes. Similar to the case with S162A, the *K_m_* supported by the S163A mutant was only moderately increased (1.7-fold) relative to wild-type in 100% PC liposomes, and was essentially the same as wild-type in 30% liposomes. Thus, the S163A mutation had a much more severe effect on *k*_cat_ than on *K_m_*. Accordingly, the reduction in catalytic efficiency exhibited by the S163A mutant was very similar to the reduction in *k*_cat_, with the *k*_cat_/*K_m_* varying from 3.2% of wild-type in 100% PC liposomes to 16% of wild-type in 30% PS liposomes. Therefore, for both mutants, increasing PS content tended to decrease the severity of the defect relative to wild-type, as exhibited by both increasing the *k*_cat_ and ameliorating the rather modest defects in *K_m_*.

**Figure 2:**
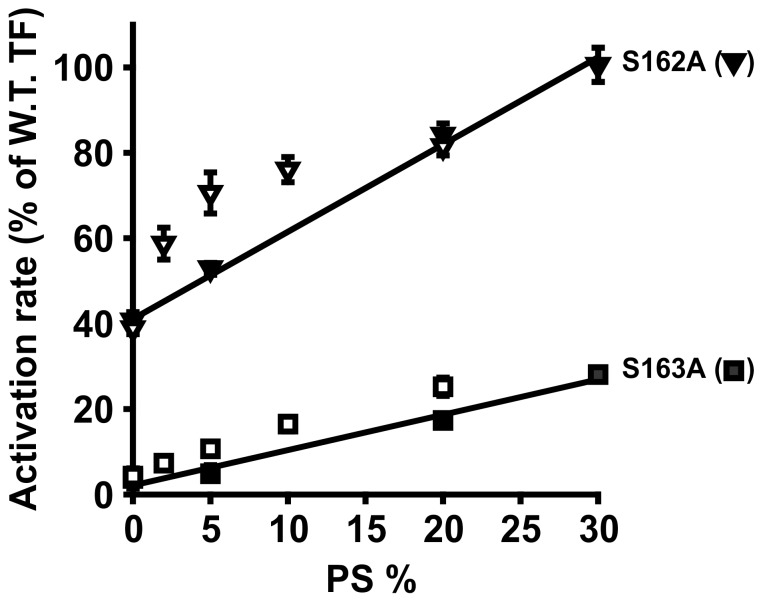
Influence of PE on FX activation by membTF mutants. Initial rates were quantified for activation of FX by FVIIa bound to membTF mutants S162A (inverted triangles) and S163A (squares) in liposomes of the indicated mol% PS in the absence (filled symbols) and presence (open symbols) of 30 mol% PE (balance = PC). Data are normalized to wild-type membTF at the same phospholipid content. Data are mean ± SD, *n = *3.

PE synergizes with low levels of PS to support membrane-dependent clotting reactions, including FX activation by TF/FVIIa [25]. We therefore examined initial rates of FX activation for two representative mutants (S162A and S163A) in membTF-liposomes that contained 0–20% PS with or without 30% PE (balance = PC; Figure 2). The presence of 30% PE somewhat increased relative rates of FX activation supported by these membTF mutants (especially at lower PS contents). However, the mutants were still deficient in FX activation even in the presence of PE. Thus, PE slightly enhances the ability of PS to ameliorate the deficiency of these mutants in FX activation.

All eight membTF mutants supported reduced rates of activation of the alternative protein substrate, FIX (Figure 1C and D; and Table 1). As with FX activation, increasing PS content increased the FIX activation rates of these mutants. Four (K159A, S163A, G164A and D180A) exhibited linear relationships between activity and PS content, while the other four (S160A, S162A, K165A and K166A) plateaued at 20% PS. There was reasonable agreement between our findings and previous studies of five of these mutants (Table 1), but with the same caveats about unknown phospholipid compositions in prior studies.

### Effects of TF Mutations in Plasma Clotting Assays

**Figure 3:**
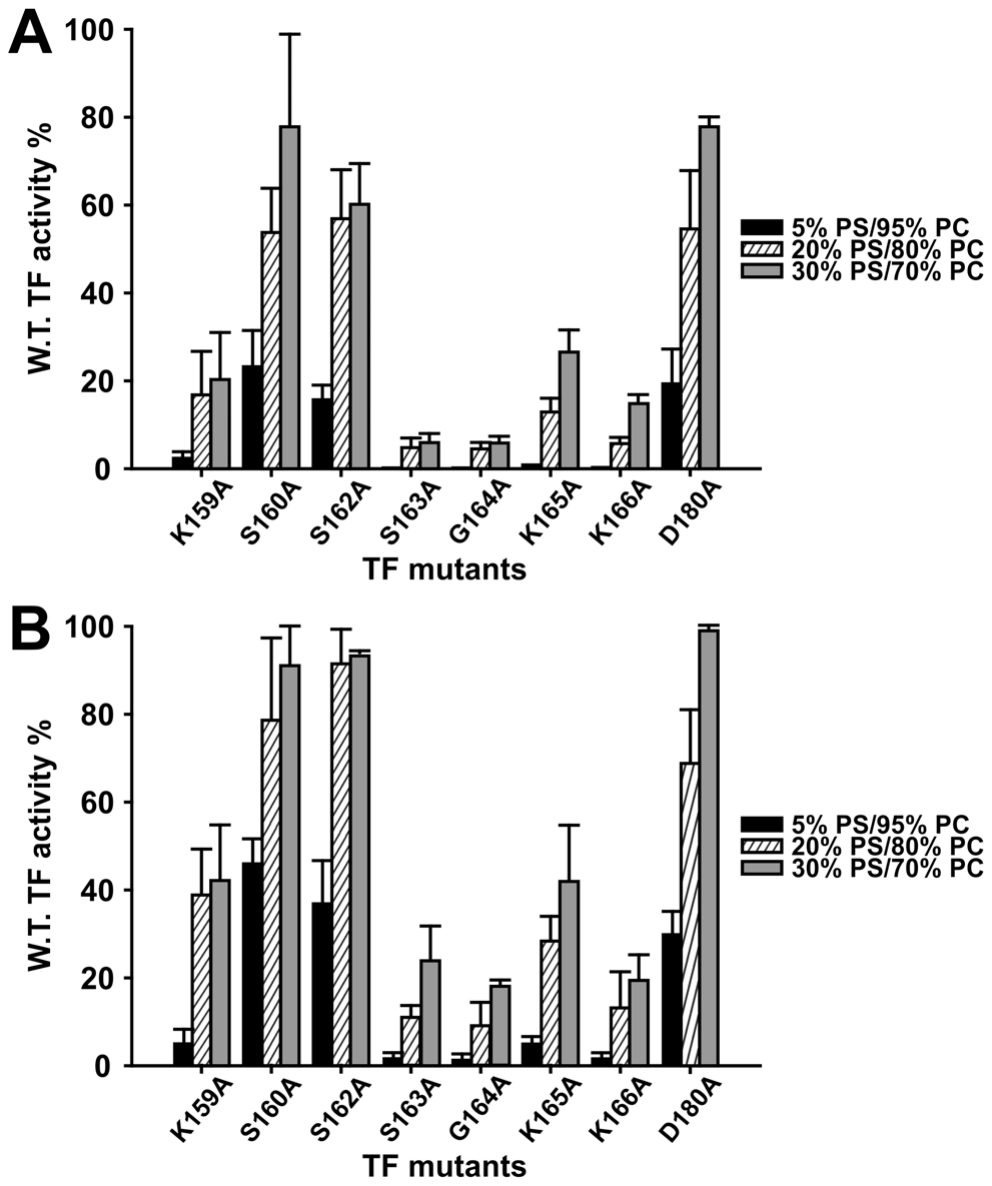
Procoagulant activities of membTF mutants. Clotting times of normal plasma, with no added FVIIa (A) or with 10 nM added FVIIa (B), were quantified in assays triggered by relipidated wild-type or mutant membTF. The membTF-liposomes contained 5% PS (*solid bars*), 20% PS (*hatched bars*) or 30% PS (*gray bars*) (balance = PC). Specific activities of mutants are plotted as percent of wild-type activity at the same phospholipid composition. Data are mean ± SD, *n = *2 or 3.

Plasma clotting assays were employed to examine the procoagulant activities of membTF mutants. By their nature, clotting assays test for additional functions of TF compared to assays with purified proteins; importantly, this includes the ability of TF to promote the conversion of zymogen FVII to FVIIa [1]. Also, clotting assays are performed under non-equilibrium conditions, so rates of assembly of protein-protein and protein-membrane complexes can impact clotting times. Accordingly, the eight membTF mutants studied in Figure 1 were incorporated into liposomes containing 5, 20 or 30% PS (balance = PC) and clotting activities were normalized to wild-type membTF at each PS content (Figure 3A). The mutants were typically more deficient in clotting activity than they were in supporting the activation of FIX or FX by purified TF/FVIIa complexes. This was especially true for 5% PS liposomes, in which all eight mutants had low activity and in which five (K159A, S163A, G164A, K165A and K166A) had little or no clotting activity. As with the assays using purified proteins, raising the PS content of membTF-liposomes substantially increased the activity of mutants relative to wild-type. The effects of seven of these mutations (all but D180A) on TF clotting activity have been reported by others, although the previously reported activities were all substantially higher than found in this study (Table 1). The very different membrane compositions used in prior studies make direct comparisons with our findings difficult.

One of the functions of TF is to promote the rapid conversion of zymogen FVII to FVIIa during clotting reactions. If this function is deficient in the mutants, it could contribute to reduced procoagulant activities since plasma contains only trace levels of pre-formed FVIIa [26]. In order to eliminate the contribution of FVII activation from clotting assays, we added 10 nM FVIIa to plasma, thus bypassing the need for TF to promote the conversion of FVII to FVIIa. Under these conditions (Figure 3B), the mutants showed a similar pattern of clotting activities compared to plasma without added FVIIa, although the relative activities in all cases were somewhat higher than when tested in the absence of added FVIIa. This finding suggests that the mutants have some deficiency in supporting the conversion of FVII to FVIIa, but that this alone cannot explain their reduced activities. As before, the relative specific procoagulant activities of the membTF mutants increased with increasing PS content in the liposomes.

### Location of Putative Lipid-interacting Residues in TF

**Figure 4:**
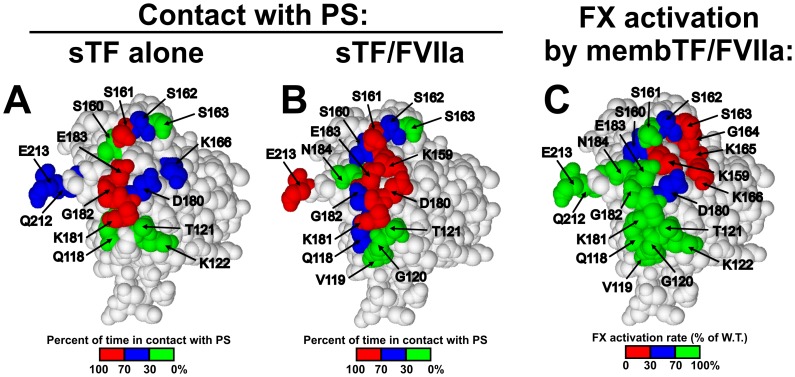
TF residues that putatively interact with PS. The crystal structure of sTF (Protein Data Bank file 1BOY [33]) is arranged so that the area proposed to interact with membrane is facing the viewer. In (A) and (B), residues are colored by percentage of time they directly interacted with PS in simulations [10]: *green*, <30% of the time; *blue*, 30–70% of the time; and *red*, >70% of the time. A) is from simulations of sTF alone on the membrane, and B) from simulations of sTF/FVIIa on the membrane. C) TF residues are colored according to their effects, when mutated to Ala in this study, on normalized FX activation rates using membTF-liposomes with 5% PS/95% PC: *red*, <30%; *blue*, 30–70%; and *green*, >70%.

The TF residues examined in this study are color-coded in the crystal structure of sTF in Figure 4A and B, according to their contact probabilities with PS in simulations [10]. In comparison, Figure 4C shows the TF residues mutated in this study, colored according to their deficiencies in FX activation. In simulations, the angle of sTF relative to the membrane surface was altered when it complexed with FVIIa [10]. This leaning motion is readily appreciated by comparing the membrane contact probabilities of residues in sTF alone (Figure 4A) or with FVIIa (Figure 4B). Further comparing these membrane footprints with the pattern of TF residues whose mutations decreased FX activation (Figure 4C), may give insights into the arrangement of TF within the TF/FVIIa/FX/membrane complex (whose structure is currently unknown). The most profound defects in FX activation were exhibited by mutations in or near the loop of TF from Lys159 to Gly164 (red in Figure 4C), which is likely to be very flexible as it lacks interpretable electron density in most crystal structures [27], [28]. This region is proposed to be an important part of the putative exosite of TF [3]–[6]. Residues Lys165 and Lys166 in particular are strongly suspected to interact directly with the substrates FX and FIX, in a manner that requires the presence of the membrane-binding domain of FX or FIX (its γ-carboxyglutamate-rich domain, or GLA domain) [29]. These TF residues lie at the very “bottom” of the C-terminal half of the TF ectodomain, and therefore should be adjacent to the membrane surface. We speculate that, by interacting directly with phospholipids, this flexible loop and the nearby exosite region are induced to undergo conformational changes that create an exosite conformation favorable for docking protein substrates like FX or IX. The mutations in our study may weaken this interaction between TF and the membrane, a deficiency that could be partially to fully overcome (depending on the precise mutation) by increasing the PS content.

### Selection of TF Mutants with Enhanced FVIIa Affinity

**Figure 5:**
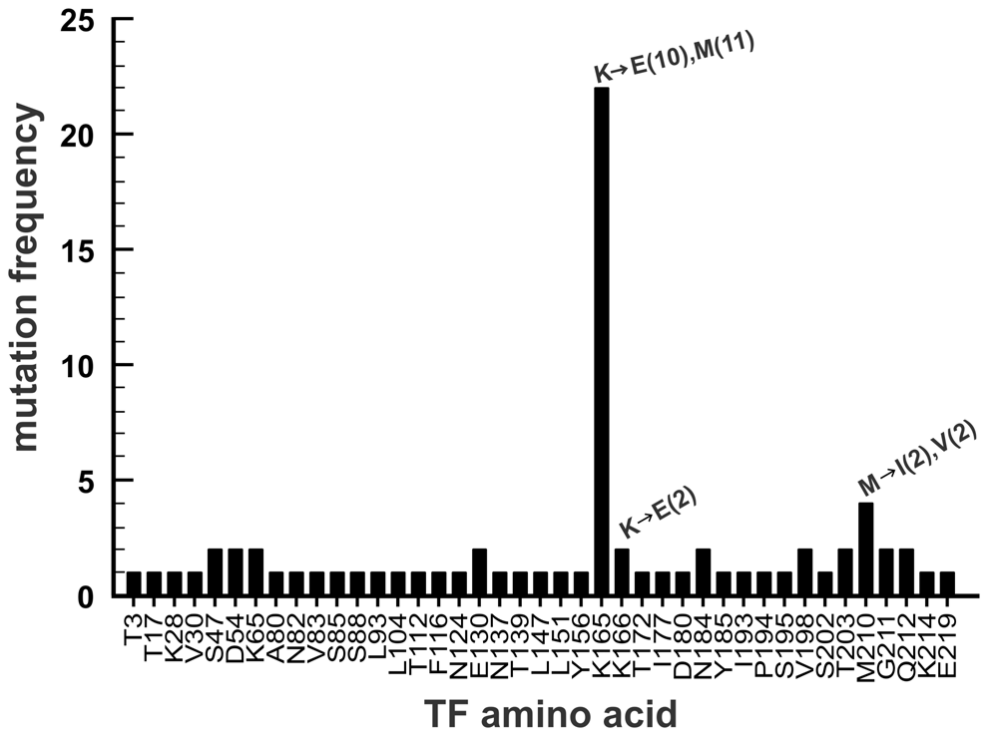
TF mutants selected by yeast surface display for enhanced FVIIa binding. After random mutagenesis, sTF mutants were expressed on the surface of yeast cells and selected for increased FVIIa binding as described in Methods. 37 clones were sequenced and the frequencies of the resulting mutations are plotted by residue number. The actual numbers of observed amino acid substitutions by amino acid type are indicated on the graph for residues K165, K166 and M210.

**Figure 6:**
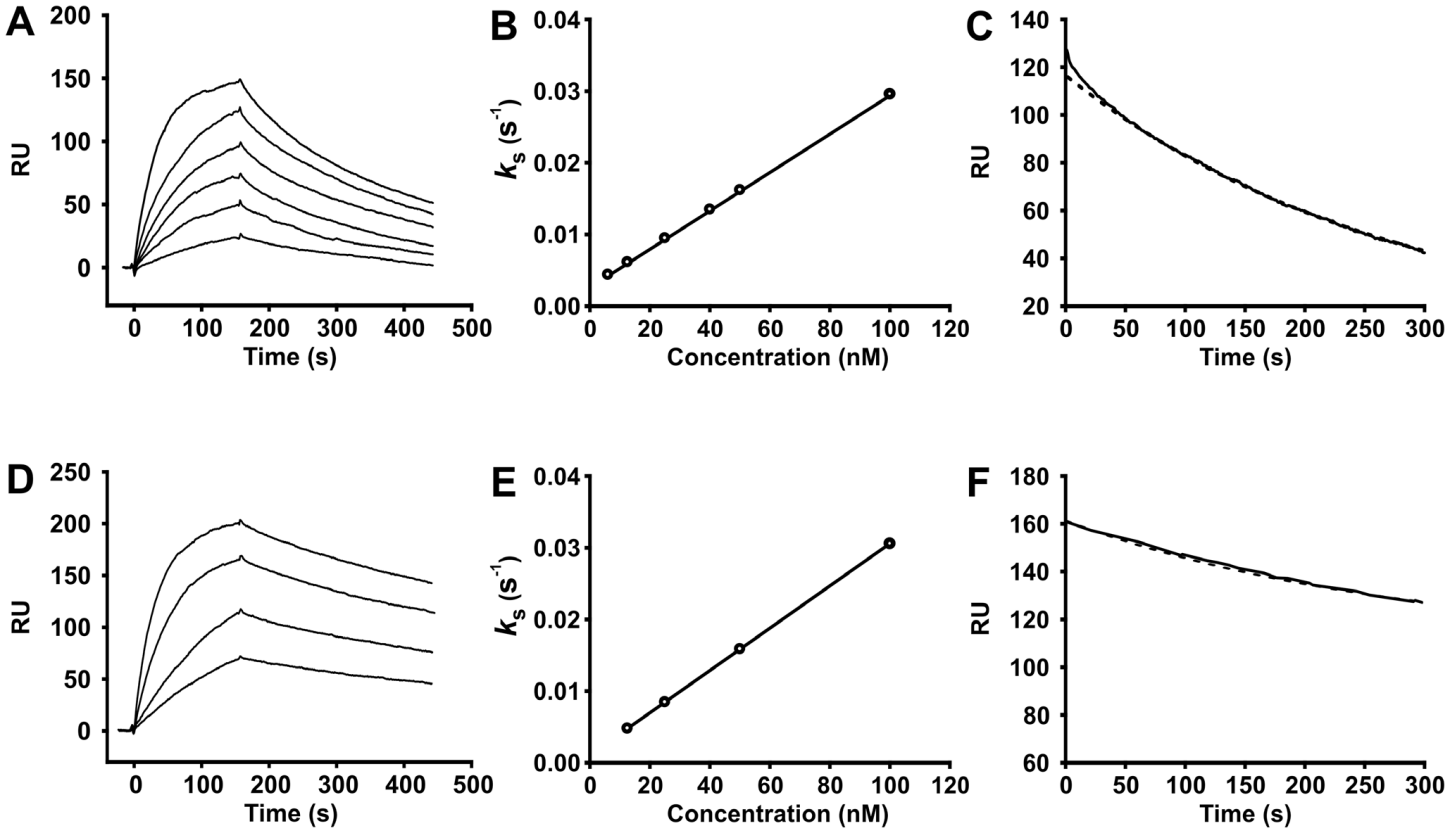
FVIIa binding to sTF. Binding of FVIIa to immobilized wild-type sTF (panels A–C) or the K165E mutant (panels D–F) were measured using SPR. (A,D) Resonance Units (RU) obtained when varying FVIIa concentrations (6, 12.5, 25, 40, 50, and 100 nM FVIIa for panel A; 12.5, 25, 50, and 100 nM FVIIa for panel D) were flowed over immobilized sTF on sensorchips. (B,E) Concentration dependence of *k*_s_ values determined by nonlinear regression analysis of the association data from panels A and D, respectively (from 0–160 s). *Solid lines* are linear least-squares fits of the equation *k*_s_ = *k*_on_C+*k*_off_, where C is the concentration of free FVIIa. (C,F) Sensorgrams (*solid lines*) observed for dissociation following injection of 50 nM FVIIa; the *dashed lines* are the result of nonlinear regression analysis using a single-exponential decay.

To further investigate the nature of allosteric interactions between TF with FVIIa, TF mutants were selected to achieve enhanced binding affinity for FVIIa. A randomly-mutated sTF library was displayed on the surface of yeast, and five cycles of flow cytometry using a relatively low *fl*-FVIIa concentration (8 nM) was used to select mutants with increased FVIIa binding. A significant enrichment in the FVIIa-positive population was observed by the fifth sorting, after which 37 yeast clones were isolated and their sTF cDNAs sequenced, resulting in 29 unique sTF DNA sequences containing a total of 75 non-silent mutations. Mutations at a single residue, Lys165, dominated the collection of sTF mutants (Figure 5). Lys165 is part of the putative substrate-binding exosite of TF [3]. We determined the effect of mutating this TF residue to Glu on FVIIa binding affinity (Figure 6). FVIIa binding to wild-type sTF had *k*_on_ = 2.68 (±0.001)×10^5^ M^−1^ s^−1^ and *k*_off_ = 3.63 (±0.12)×10^−3^ s^−1^, with a calculated *K*_d_ of 13.5±0.4 nM. The sTF mutant K165E had an enhanced binding affinity, with similar *k*_on_ (2.95 (±0.003)×10^5^ M^−1^ s^−1^) but slower *k*_off_ (1.29 (±0.007)×10^−3^ s^−1^) and lower *K*_d_ (4.4±0.02 nM). The enhanced FVIIa binding affinity for the K165E mutant is consistent with our previous study which showed enhanced binding affinity for the K165E,K166E double mutant relative to wild-type sTF [29]. Interestingly, however, mutating these two Lys residues to Ala was without effect on FVIIa binding affinity [29], [30]. We also note that our previous study showed that mutating Lys165 to Glu resulted in a significantly greater loss of TF procoagulant activity than did mutating this residue to Ala [29].

The dominance of two amino acid substitutions at a single TF residue following selection for increased FVIIa binding was surprising, especially coupled with the fact that K165 was one of the first residues whose chemical modification identified the exosite region of TF [30]. This residue is also highly conserved among mammalian TF sequences. One can speculate that there is an energy penalty associated with K165 when TF binds to FVIIa, which is ameliorated when this residue is mutated to Glu. We speculate that this energy penalty is coupled to a conformational change in the adjacent exosite region of TF. Thus, mutating K165 to Glu may simultaneously increase the affinity of sTF for FVIIa and diminish the function of the TF exosite region, *via* disabling the proposed FVIIa binding-induced conformational change in TF.

In conclusion, we found that increasing the PS content of membTF-liposomes partially or completely overcame the decreased TF activity of mutations in or near the exosite region, possibly by stabilizing the interaction of this part of TF with the membrane surface and thereby optimizing the interaction of the TF/FVIIa complex with FIX and FX. This finding is similar to previous findings that the deleterious effects of mutations of TF exosite residues K165 and K166 can also be overcome, to some extent, by increasing the PS content of TF-liposomes [29], [31]. Thus, we postulate an allosteric linkage between the interaction of TF with PS and the conformation of the nearby TF exosite. The ability of increasing the PS content of TF-liposomes to restore the activity of the TF/FVIIa complex in our mutants may be due to multiple, relatively weak phospholipid binding interactions with the TF ectodomain. For this reason, mutations which decrease the affinity of this part of TF for the membrane bilayer may require higher concentrations of PS in liposomes to saturate the binding interactions between the TF ectodomain and PS headgroups. Other explanations are also possible, including effects of elevated PS on the GLA domains of the enzyme (FVIIa) and/or its protein substrates (FIX and FX) to compensate for the effects of the TF mutants. It should be pointed out that the putative exosite region of TF has, to date, been characterized only via mutagenesis studies. Thus, to our knowledge, binding of FIX or FX to this or any other region of TF has not been demonstrated experimentally. Direct physical studies of the TF/FVIIa/FIX or TF/FVIIa/FIX complexes, possibly including solving crystal structures of such trimolecular complexes but more preferably studies conducted in the context of their assembly on PS-containing membrane bilayers, are needed to address these questions in more detail.
